# Reasons for premature removal of progestogen implant among women in a community in Pretoria

**DOI:** 10.4102/jcmsa.v2i1.92

**Published:** 2024-08-28

**Authors:** Wanda M. Moeletsi, Suzan L.N. Nyalunga, Margaret M.M. Ramochele, Tombo Bongongo

**Affiliations:** 1 Department of Family Medicine and Primary Health Care, School of Medicine, Sefako Makgatho Health Sciences University, Pretoria, South Africa

**Keywords:** women’s reasons, premature removal, implanon, Temba CHC, Pretoria community

## Abstract

**Background:**

Implanon is widely used in South Africa and around the world. However, a significant number of users discontinue it prematurely. The current study aims to explore the reasons for women’s early removal of the progestogen implant, known as Implanon, in a Pretoria community.

**Methods:**

This was a qualitative study applying a descriptive phenomenological design, with nine one-on-one interviews done at Temba Community Health Centre among women who had their Implanon implants removed sooner than the intended length of time.

**Results:**

Four major themes emerged from the data collection: reasons for choosing Implanon, side effects of Implanon, the social impact of Implanon and public attitudes towards Implanon. These four themes were further explored through 13 subthemes.

**Conclusion:**

The participants acknowledged and valued the benefits of Implanon, but they also encountered undesirable side effects such headaches, bleeding, breast pain and enlargement, decreased libido, and mood swings. The primary causes of the implant’s early removal were these side effects, among which bleeding was the most frequently reported problem.

**Contribution:**

Implanon-induced bleeding has an impact on the economics of households and the sexual lives of women, as it requires the purchasing of sanitary products and other medications.

## Introduction

Long-acting reversible contraceptives are contraceptive methods that require administration less than once every cycle or month.^1^ They include copper intrauterine devices, progestogen-only intrauterine systems, progestogen-only injectable contraceptives and progestogen-only subdermal implants.^1^ These contraceptives, such as Implanon, a progestogen-only subdermal implant, have been championed as highly effective contraceptives, regarded as suitable to decrease the rates of unwanted pregnancies and frequently viewed as especially suitable for use at all life stages of women.^1,2^ Of the long-acting reversible contraceptives, the subdermal contraceptive implant Implanon is the most used method in the world.^1^ Implanon is a single-rod contraceptive progesterone implant measuring 4.0 cm in length and 2.0 mm in diameter. It is implanted in a woman’s non-dominant upper arm to provide contraceptive protection for 3 years. It is a non-biodegradable implant that contains 68 mg of etonogestrel (progestogen) in an ethylene vinyl acetate (EVA) copolymer core, surrounded by an EVA membrane. Each rod consists of an inner core containing 60% etonogestrel and 40% EVA and an outer membrane containing 100% EVA.^3^ There is no accumulation of EVA; it is excreted 60% in urine and 40% in faeces. The mechanism of action of the progestogen implant is ovulation inhibition and an increase in the viscosity of cervical mucus.^3^

Every year, around 14 million unintended pregnancies occur in sub-Saharan Africa, with a considerable proportion caused by poor use of hormonal contraception.^4^ This includes the early removal of the Implanon contraceptive, which might be an example of the mentioned concern in the region.^4^ This includes early removal of the Implanon contraceptive as one of the most significant issues in the family planning programme in the region. In Ethiopia, for example, particularly in the district of Kucha in 2020, a qualitative study aimed at determining the prevalence and reasons for early Implanon removal discovered that 9.2% were due to poor counselling, 0.1% due to fear of side effects and 5.2% due to poor satisfaction with the health service. Again, early removal of the Implanon contraceptive constituted the most significant source of concern for family planning programmes in the country.^4^ In Kampala, Uganda, looking at the same aim (Implanon prevalence and causes of early discontinuation), of the 275 participants, 51% had menstruation irregularities, 20% had headaches, 16% had body weight changes, 8% had arm discomfort and 12% had lost their sexual interest. About 47% of participants also said lack of counselling options and living in rural areas were the primary factors for the removal of Implanon. As a result, the authors advocate addressing the high rate of early contraceptive removal by strengthening counselling services about potential negative effects.^5^

In 2014, a South African qualitative study on subdermal Implanon progestogen implants focussed on the reasons given by women who removed their Implanon prematurely.^6^ The main issues given for the device’s early removal included undesirable side effects such as menstrual problems, arm discomfort (where the Implanon was inserted) and weight gain, in addition to family and social influences.^6^ In 2014, South Africa implemented the Implanon progestogen implant to broaden the selection of contraceptive methods available in the public sector, with a particular emphasis on long-acting reversible methods.^7^ The introduction of Implanon in South Africa was aimed at minimising the high rate of unintended pregnancies. Although this method was accepted with much excitement at the beginning, many women eventually removed their Implanon implants before the expiry date. Among those who discontinued the implant, reasons for early removal were cited as deficiencies in counselling around the effectiveness and side effects. Side effects included headaches, weight gain, hair loss and acne, among others.^8^

Studies have been undertaken across South Africa on the reasons for early Implanon removal, but many of them were quantitative. The current study, while focussed on the same goal, employed a qualitative approach to fill the gap by giving an in-depth analysis of the phenomenon. Therefore, this study explored the reasons for early removal of the progestogen implant Implanon among women in a Pretoria community.

## Research methods and design

### Study design

This was a qualitative study using a descriptive phenomenological design. Nine one-to-one interviews were conducted to explore women’s reasons for early removal of the progestogen implant Implanon in a Pretoria community.

### Study setting

The study was conducted at Temba Community Health Centre (CHC), part of Tshwane Primary Health Care District. It provides primary and preventative healthcare services for the community. The CHC is situated 51 km to the north of Pretoria (Tshwane), in Gauteng province. Temba is an area in the small town of Hammanskraal, which has a population of 94 273 people, 61 138 (64.88%) of whom are Africans who speak Setswana as their primary language, according to a 2011 South African statistics or census report.^9,10^

### Study population, sampling technique and sample size

The study population consisted of women who removed their progestogen implants before the expiration of the 3-year insertion period at Temba CHC between 01 July 2015 and 01 July 2018. According to the family planning unit registry of the CHC, 15 women were identified and contacted telephonically, with only nine responding to the call. Purposive sampling was used to select the participants and the information from the participants reached saturation at the ninth participant.

### Data collection

All nine participants who responded to the call were invited to the CHC over the phone and subsequently met with the research assistant (RA) to learn more about the study. They agreed on the dates and times of the interviews. During each appointment, the RA fully addressed the research’s objectives and aim with every participant and those who agreed to participate signed an informed permission form before they were interviewed. This occurred from 01 July 2018 to 01 October 2018, at Temba CHC, and interviews lasted between 40 min and 50 min, depending on the date and time chosen by the participant.

The data were collected by the RA, a retired nurse with extensive expertise in conducting qualitative interviews and fluency in English and Setswana, the two languages spoken in the study area.

Before data collection, the tool (interview guide) was pre-tested on three clients who did not form part of the study sample; this took place at Phedisong CHC, which is located almost 63 km away from the Temba CHC. This ensured the non-contamination of data, while the two areas have people with similar cultures, languages, food habits and so on. The results assisted the author in confirming that the interview guide intended to ask about the study’s objectives. The interviews were conducted by the RA on the appointed date and time for each participant, and a semi-structured interview guide with open-ended questions was used. While defining and outlining the necessary competencies for planning and carrying out semi-structured interviews in family medicine and primary care research contexts, DE Jonckheere and Vaughn^11^ pointed out that the approach enables the researcher to gather unstructured data investigate participant ideas, attitudes and opinions regarding a specific subject and probe deeply into private and occasionally delicate matters.^11^ Semi-structured interviews consist of several key questions that not only help to define the areas to be explored but also allow the interviewee to diverge in order to pursue an idea or response in more detail.^12^

### Interview guide

English and Setswana interview guides were used. The English interview guide was translated into Setswana. Tswana is the most spoken language in Hammanskraal, and most inhabitants also understand English. From the interview guide, one exploratory question (English version) was used, namely: ‘What influenced your choice of early removal of the progestogen implant?’, followed by prompting questions being put to the participants. The Setswana version was as follows: ‘*Ke eng se se go tlhotlheleditseng go ntsha Implanon pele ga nako?*’. The answers provided by the participants were written down in a notebook and recorded on an audio tape. Baseline characteristics such as age, educational level, employment, marital status, number of children and history of abortions were also recorded to link them to the interviews and for data analysis. The audio-tape interviews were transcribed verbatim and recorded in a notebook. Transcribed data in the vernacular was translated into English by a linguistic expert.

### Data analysis

A trained qualitative data analysis RA used Microsoft Word to transcribe all the material gathered from participants. The RA evaluated the typed transcripts and gave them to the principal researcher to ensure they were correct. The transcripts were processed using Braun and Clarke’s thematic analysis method.^13^ The following different steps were used for data analysis: (1) familiarity with the study data: after the transcribing was completed, the researcher and RA read and reread the data to check the accuracy of the information collected from participants; (2) generate codes: the participants were identified as P1, P2, P3, P4, P5, P6, P7, P8 and P9, and each piece of information was linked to the participant who volunteered it; (3) searching for themes: checking to see if the themes fit with the coded extracts and creating a thematic map of the study; (4) review of themes; (5) define and name topics: each theme was identified and named and (6) creating a report for final analysis.

### Ethical considerations

The Operational manager of Temba CHC authorised the study to be done at the facility, while Sefako Makgatho University Research Ethics Committee (SMUREC) granted a clearance certificate (SMUREC/M/83/2018: PG). Confidentiality was applied (no identification elements such as names, ID numbers or addresses were required) during the study process.

## Results

Nine participants were interviewed in this study. Table 1 illustrates the demographic features of the women who participated in the study. The oldest was 27 years of age, most were single, the highest educational level attained was tertiary, while the greatest number of children was two, with most participants being unemployed and three of them having miscarried.

**TABLE 1:** Demographic characteristics of the participants.

Participants	Age (years)	Marital status	Education level	Number of children	Employed	Miscarriage
P1	27	M	Tertiary	2	No	No
P2	27	S	Tertiary	1	Yes	No
P3	22	S	Tertiary	0	No	No
P4	21	S	Matric	0	No	Yes
P5	32	S	Matric	2	No	No
P6	18	S	Grade 10	0	No	No
P7	21	S	Tertiary	1	No	No
P8	20	S	Matric	1	Yes	Yes
P9	26	M	Matric	2	Yes	Yes

M, married; S, single.

A summary of the sociodemographic information is presented in Table 2. The participants had an average age of 24 years. Out of the sampled population, 22% had previously been married, while 33% were employed, 75% had been pregnant previously and had children and 33% of them had miscarried.

**TABLE 2:** Summary of the sociodemographic data.

Variable	Mean age (years)	Pregnant before	Married before	Employed	Have children	Miscarried
Number	24	7	2	3	6	3
%	-	77	22	33	66	33

### Themes and subthemes

Four themes emerged from the data: (1) the reason for the decision to use Implanon, (2) side effects of Implanon, (3) social impact of Implanon and (4) public perception of the use of Implanon. A further 13 subthemes emerged, as seen in Table 3.

**TABLE 3:** Themes and subthemes.

Themes		Subthemes
1.	Reason for choosing the progestogen implant	Initial motivation to choose Implanon.
2.	Side effects of progestogen implant	2.1. Menstrual side effects Prolonged and irregular bleeding 2.2. Non-menstrual side effects Weight gain Acne Hair loss Breast pain and enlargement Headaches 2.3. Sexual dysfunction side effects Decreased libido and vaginal dryness 2.4. Psychological side effects Mood swings
3.	Social impact of progestogen implant	3.1. Strained relationship due to challenging sexual relations and appearance 3.2. Excessive spending on sanitary and feminine products 3.3. Spending money on treating side effects
4.	Societal perceptions of the use of Implanon	Risk of infertility

#### Theme 1: Reason for choosing the progestogen implant

Women often face decisions related to family planning. This is because, most of the time, they bear more responsibility for childcare than men do. When asked why they chose to use Implanon, participants gave many explanations. The answers seem to be determined by the participant’s life stage. What stood out was that the main reason was to avoid unplanned pregnancies in order to achieve personal goals before conceiving and committing to caring for a child. Those of employable age stated that they picked it because they were employed, while participants without employment cited financial difficulties. Those who were studying indicated a desire to finish their degrees as reasons for selecting Implanon. Another reason for opting for Implanon compared to other methods of contraception was the long-time frame of effectiveness, with minimal maintenance of the implant:

‘Because I want to delay having babies because I am not working.’ (P4, 21 years old, Single, Matric)

‘Because I don’t want to have a baby now. I want to finish my studies.’ (P3, 22 years old, Single, Tertiary)

‘We decided on Implanon because we wanted to prevent pregnancy so that we save money because of the situation I found myself in.’ (P9, 26 years old, Married, Matric)

‘My experience with Implanon is that it is good for school-going girls, truly because one does not go to the clinic now and then.’ (P6, 18 years old, Single, Grade 10)

‘This device is good but … I was having some problems but it’s good because it lasts for three years and then due to overcrowding at the clinic it saves time.’ (P8, 20 years old, Single, Matric)

#### Theme 2: Side effects

**Subtheme 2.1: Menstrual side effects:** Prolonged and heavy bleeding: Progestogen implant (Implanon) users at Temba CHC reported excessive and prolonged menstrual bleeding. Some women reported short but frequent bleeding:

‘The bad effects for me are because I was having bleeding throughout since using this device.’ (P2, 27 years old, Single, Tertiary)

‘I was happy about it until I notice that I am having heavy bleeding ever since I inserted it.’ (P1, 27 years old, Married, Tertiary)

‘The bad effects for me are because I was bleeding throughout since using this device. No, it is only the bleeding. Yeah, because I came to the clinic, and they gave me some pills to stop the bleeding, but it continues that is why I decided to remove it.’ (P8, 20 years old, Single, Matric)

**Subtheme 2.2: Non-menstrual side effects:** Weight gain, hair loss and acne: Some participants complained about non-menstrual side effects that affected them, such as weight gain, which was noticed after the insertion of the Implanon.

Some participants noticed acne and hair loss as they started using Implanon. These participants cited these side effects as reasons for deciding to remove the Implanon:

‘It makes my menstruation prolonged, I am losing my hair, it gives me headaches, and I gain weight. I don’t like the way I have become.’ (P7, 21 years old, Single, Tertiary)

‘They also see changes in me, everyone is telling me how I gained weight.’ (P1, 27 years old, Married, Tertiary)

‘It affected how I looked at myself because people comment on my skin, especially the acne that I developed, and it affected me a lot.’ (P2, 27 years old, Single, Tertiary)

Some participants reported only a few negative effects, while others reported a wide range of side effects from the Implanon. These adverse effects would have an impact on a participant’s self-esteem because they involve visible features. Weight gain, hair loss and acne were among the negative effects that participants reported as affecting their self-perception.

**Headaches and breast pain and enlargement:** Moreover, participants reported side effects that were related to being made uncomfortable by pain. Some reported constant or recurrent headaches as well as breast pain and enlargement:

‘And then also it gives me a lot of headaches.’ (P5, 32 years old, Single, Matric)

‘I have a light headache. I struggle a bit with headaches and heavy periods, which I never had before. After I put the implant, I also saw that my breasts were becoming big and painful, I thought it will go away, but it didn’t.’ (P6, 18 years old, Single, Grade 10)

Some participants experienced these side effects and were convinced that they were caused by the implant because they felt better and the side effects went away once the implant was removed.

**Subtheme 2.3: Sexual dysfunction side effects:** Decreased libido and vaginal dryness: Participants reported experiencing side effects related to their sexual health. Some of them reported a decrease in libido, while others received feedback on this from their partners:

‘My partner says that I do not want to have sex since I put in the implant.’ (P4, 21 years old, Single, Matric)

‘I removed it for the reason of saving my marriage, because I could lose my marriage if I do not feel like having sex all the time.’ (P9, 26 years old, Married, Matric)

‘I did not want to have sex because of the dryness that I was experiencing in my private parts. Sex was not enjoyable. So, I remove it.’ (P6, 18 years old, Single, Grade 10)

**Subtheme 2.4: Psychological side effects:** Mood swings: Participants reflected on the emotional impact of the side effects that they experienced after they inserted the implant. Participants reported feeling irritable most of the time and finding that their moods fluctuated:

‘It was the skin problems and the mood swings.’ (P2, 27 years old, Single, Tertiary)

‘I was heavily bleeding most of the time, which was not nice, and I also saw that I am an angry person. I would shout at the children and get angry about very small things at home. Even my husband said so.’ (P5, 32 years old, Single, Matric)

Some participants were able to notice the change in their moods and emotions when they were using Implanon compared to before they inserted the implant. They were aware that the moods impacted not only themselves but their families too.

#### Theme 3: Social impact of the progestogen implant

Subtheme 3.1. Strained relationships: The use of Implanon also influenced the participants’ intimate relationships. The heavy bleeding and the low libido as side effects of the Implanon led to constant fights between the participants and their spouses or partners. This influenced their decision to remove the implant prematurely so that they could have a healthy sexual relationship with their partner:

‘It affected me because you know as a woman if you are menstruating every day, how are you going to have sexual intercourse with your partner? So, it was a problem.’ (P3, 22 years old, Single, Tertiary)

‘Yeah, I was always fighting with my boyfriend, and he used to tell me that he will go for another girl because I am always bleeding.’ (P8, 20 years old, Single, Matric)

‘Eh, relations with the husband, he said the implant seems to be problematic to me because we no longer have intimate relations, it is a problem. … he said, I must remove it because it is bringing problems, and it will destroy my marriage. So, go back to the one you were using monthly or [*every*] two months or three months.’ (P7, 21 years old, Single, Tertiary)

Subtheme 3.2. Excessive spending on sanitary and feminine products: One of the other reasons cited for the premature removal of the implant was that the participants experienced heavy bleeding. This excessive bleeding increased the expenditure on sanitary and feminine products:

‘The bleeding, it was bad because I used to buy pads time and again. Yeah. I do not have money to buy pads more than once in a month.’ (P3, 22 years old, Single, Tertiary)

The findings show that there was a relationship between the financial cost of buying pads caused by heavy bleeding during the menstrual cycle and the premature removal of the progestogen implant (Implanon). The financial burden is also implicated in tension felt in the relationship with partners. Financial challenges will impact any relationship. The participants reported that the excessive spending on sanitary and feminine products was having an impact on the household’s budget. The budgetary challenges affected their relationships with family members, especially partners and husbands.

‘It is costing our household money. It is causing fights with my husband.’ (P5, 32 years old, Single, Matric)

Subtheme 3.3. Spending money on treating side effects: The financial challenges seemed to extend to the family and household budget, which further contributed to the tension and heated emotional atmosphere between participants and their spouses. The financial impact included needing to treat other side effects, such as constant headaches, hair loss and acne. Participants felt that they would rather remove the implant instead of putting up with the side effects and related expenditures:

‘I’m buying further acne medications, and paracetamol with my pocket money.’ (P4, 21 years old, Single, Matric)

#### Theme 4: Societal perceptions of the use of Implanon

Some participants did not have extensive side effects, but their decision to remove the Implanon was impacted by conversations they had with other people. They heard from other people in the community that the implant may hinder one from conceiving at a later stage. This led them to remove the implant rather than risk infertility:

‘For my side, I don’t have a problem, but the only thing is that I heard people complaining that if I want babies, I won’t get one in the future, so that’s why I am scared, because I don’t have babies.’ (P4, 21 years old, Single, Matric)

‘Because if you insert the Implanon it’s once and you remove it after three Years, but unfortunately, you will not have any babies because of the implant. After I heard the comments from other people, then I became scared.’ (P6, 18 years old, Single, Grade 10)

Societal perceptions can be powerful motivators of behaviour. When an individual gets information from persons who are considered to know more about fertility and what can negatively impact it, they will accept the premise without confirming the information.

## Discussion

Implanon is widely used in South Africa and worldwide.^1,2,14,15^ The duration of use is 3 years, and it has no effect on a woman’s fertility. Women who take this sort of contraception see an immediate return to fertility.^14^ Participants in this study were asked why they chose the implant for contraception and many stated that what drew them in was the duration of use. The participants reflected on the possible long queues at healthcare facilities if they had to attend more often, which was undesirable to them. Implanon is a highly convoluted medication used in South Africa that requires user cooperation.^15^ It has been shown to be effective for a wide range of women at various periods of their lives, including breastfeeding mothers with oestrogen contraindications, and women with diabetes, hypertension, sickle cell anaemia and HIV infection.^8,16^

The participants wished to avoid unplanned pregnancies for various reasons. The use of contraceptive methods is accepted in countries such as South Africa, where approximately 68% of women are using a contraceptive method. This increase in contraceptive acceptance should contribute to reducing the number of pregnancies from 1.3 million per year in 2014 to 1 million in 2030.^17,18,19^ Some of the participants were employed and aimed to reach certain career and life goals, while others were motivated by financial needs to prevent conception. Implanon is an effective form of contraception for preventing conception, as its mechanism of action is to prevent ovulation, thicken cervical mucus and prevent sperm penetration.^19^ Choosing Implanon was an informed decision made by the participants. They understood its efficacy and its advantages over other contraception methods.

According to the journal South African Family Practice, the health profession has seen a steady increase in Implanon discontinuation.^15^ It was recorded that 43% of women discontinued Implanon before completion of the 3 years.^15^ In this study, while participants understood the efficacy of Implanon, they removed their implants prematurely for various reasons. Removing the implant poses the risk of unwanted pregnancies for the women.^15^ According to Habte et al.,^15^ unwanted pregnancies are rife and account for 41% of the 208 million pregnancies that occur yearly. These unwanted and unplanned pregnancies happen due to lack of contraception use, with further risk of the pregnancies ending in abortion or stillbirth.^15,20^

Participants cited Implanon’s side effects as a primary factor for discontinuing this contraception option. Many of the participants cited excessive bleeding as a primary reason for removing the implant, and this heavy bleeding impacted a few facets of their lives. Literature concurs that heavy bleeding is the main side effect that women experience with the implant.^8^ According to Karlsson et al.,^21^ heavy bleeding affects general life planning, energy levels, mood levels and general physical health. In fact, women who have heavy bleeding have a lower quality of life than those who do not.^22^ The participants’ intimate relationships were adversely affected by the heavy bleeding, which also placed a financial burden on their family budget.

Participants reported non-menstrual side effects such as weight gain and acne as reasons to prematurely remove their implant. In a study carried out in the same clinic (Temba CHC) and published in 2022, weight gain was experienced by 11% of the participants.^23^ Participants also included headaches and breast pain and enlargement as factors that led to their request to remove the implant prematurely.^22^ The same side effects have been mentioned in other studies in the literature.^8,12,15^ These symptoms can be seen as hormonal symptoms that will affect a woman’s life.

Participants reported low libido due to the implant, which led to decreased interest in sexual activities, with their partners also expressing this concern. Martell et al.^23^ noted that low libido is common in women who are using contraception in general.^23^ In a study conducted by Beesham et al.,^14^ loss of libido was referred to as a reason for requesting removal of the implant at a clinic in KwaZulu-Natal. The Diagnostic and Statistical Manual of Mental Disorders: DSM-5 explains sexual dysfunction as characterised by a clinically significant disturbance in a person’s ability to respond sexually or to experience sexual pleasure.^24^ In contrast, sexual health includes many facets of a woman’s life, including physical and mental well-being, and even social circumstances. Low socioeconomic status could have an impact on women’s desire and sexual responsiveness.^20^ In this study, all these factors were seemingly affected by the Implanon.

In addition, participants mentioned that a change in mood and affect were side effects that motivated them to have the implant removed. According to Martell et al.,^23^ mood changes were among the most common perceived side effects of hormonal contraception in general. The study highlighted the importance of psychological side effects, which should not be downplayed. These authors suggested that healthcare workers should consider psychiatric and mood lability history when conducting contraceptive counselling and prescribing contraception. Mood-related symptoms should also be explored at any follow-up appointments. Mood changes were cited as a side effect of Implanon.^13^

While contraception is mostly considered by women, literature shows that women appreciate the participation of their partners concerning contraception.^25^ This includes a willingness to receive information about different contraceptive methods. In the study by Aziz et al.,^25^ a husband’s level of education was a marker for understanding and supporting a wife’s decision for choosing contraception, however, fueled by the heavy bleeding that impacted their intimate sexual lives. Partners were unsatisfied with the impact of the Implanon and, as a result, suggested that their partners remove the implant – at times even threatening to look for other sexual partners. According to Beesham et al.^14^ and Ssebatta et al.,^5^ pressure from spouses was often mentioned as a reason for discontinuing contraception. The participants in this study further highlighted the financial burden caused by the procurement of sanitary products, due to the heavy bleeding that was experienced. This financial burden was further reason for disagreements with their partners, as well as impacting the family budget.

Social perceptions are important determinants and motivators of behaviour. The participants in this study mentioned that one of the reasons for removing the implant prematurely was due to information shared in the community. Participants feared that Implanon insertion may hinder future fertility, prompting them to remove the implant. Potgieter et al.^26^ emphasise the importance of public perception in decision-making regarding contraception, urging against underestimating its significance. In addition, when women experience adverse effects, additional assistance is required.^27^ Similarly, Moeti et al.^22^ agree that community has a major influence on women’s perceptions regarding contraception. Beesham et al.^14^ found media influence and community misinformation affect early implant removal, emphasising the need for healthcare providers to address misconceptions and confusion in societal spaces.^8,26^

## Conclusion

Despite the benefits of Implanon, which the participants recognised and valued, undesirable side effects such as bleeding, headaches, breast pain and enlargement, decreased libido and mood changes were the reasons for the implant’s early removal, with bleeding being the most reported.
